# Development of Poly(methyl methacrylate)/nano-hydroxyapatite (PMMA/nHA) Nanofibers for Tissue Engineering Regeneration Using an Electrospinning Technique

**DOI:** 10.3390/polym16040531

**Published:** 2024-02-16

**Authors:** Angelika Zaszczyńska, Dorota Kołbuk, Arkadiusz Gradys, Paweł Sajkiewicz

**Affiliations:** Laboratory of Polymers & Biomaterials, Institute of Fundamental Technological Research, Polish Academy of Sciences, Pawinskiego 5b St., 02-106 Warsaw, Poland; azasz@ippt.pan.pl (A.Z.); dkolbuk@ippt.pan.pl (D.K.); argrad@ippt.pan.pl (A.G.)

**Keywords:** biomaterials, nanofibrous scaffolds, bone tissue engineering

## Abstract

The study explores the in vitro biocompatibility and osteoconductivity of poly(methyl methacrylate)/nano-hydroxyapatite (PMMA/nHA) composite nanofibrous scaffolds for bone tissue engineering (BTE). Electrospun scaffolds, exhibiting both low and high fiber orientation, were investigated. The inclusion of hydroxyapatite nanoparticles enhances the osteoconductivity of the scaffolds while maintaining the ease of fabrication through electrospinning. SEM analysis confirms the high-quality morphology of the scaffolds, with successful incorporation of nHA evidenced by SEM-EDS and FTIR methods. DSC analysis indicates that nHA addition increases the PMMA glass transition temperature (Tg) and reduces stress relaxation during electrospinning. Furthermore, higher fiber orientation affects PMMA Tg and stress relaxation differently. Biological studies demonstrate the composite material’s non-toxicity, excellent osteoblast viability, attachment, spreading, and proliferation. Overall, PMMA/nHA composite scaffolds show promise for BTE applications.

## 1. Introduction

Tissue engineering, especially bone reconstruction, has been extensively investigated for many years [1,2]. Bone defects that arise from causes such as severe trauma, infection, tumor removal, or nonunion after fracture are often problematic to heal. They may cause bone deformity, including inadequate bone length [3]. Such a situation leads to disturbances in the structure or function of the bones, and infection may lead to the patient’s death [4]. The gold standard treatment is the use of the autograft due to its high compatibility with the cells in the patient’s body. Nevertheless, the main disadvantages of this method are donor-site morbidity and various structures of bone from different parts of the body. In effect, scientists try to create alternative transplant methods [5,6].

Current medicine attempts to meet these requirements. One of the more studied specific methods is the electrospinning technique [7,8]. Electrospinning is a popular technique for producing micro- and nanofibers that have received scientific and industrial attention [9]. Highly porous scaffolds with small pore sizes and high surface-to-volume ratios are widely used. Three-dimensional structures with aligned and random fiber orientations can be formed using different types of collectors. Improvements in electrospinning for biomedical applications are constantly proceeding, and bicomponent fibers; multijet electrospinning; needleless electrospinning in the case of scale-up; emulsion electrospinning; surface energy-free electrospinning; and multifluid electrospinning including coaxial, triaxial, and side-by-side electrospinning have been explored [10,11,12].

**Figure 1:**
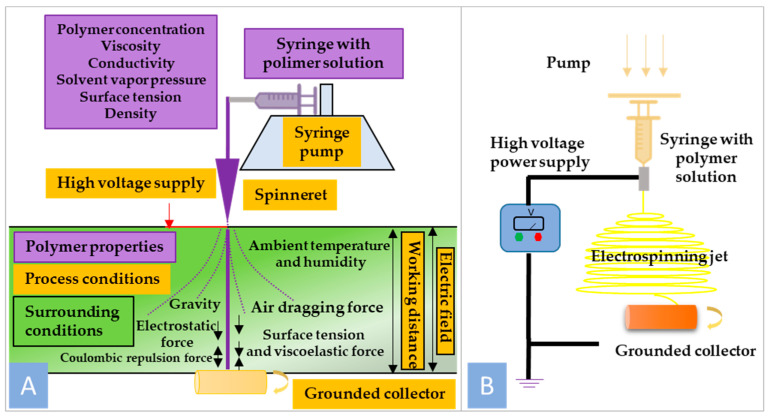
(**A**) General scheme of nanofiber formation using electrospinning technique; (**B**) schematic illustration of electrospinning setup.

The electrospinning setup consists of a high-voltage power supply, a spinneret section, and a grounded collector. A typical spinneret section consists of a syringe ending with a needle containing polymer solution, which is supplied at a selected constant flow rate by a precise pump. High voltage is applied to the tip of the metal needle, creating Coulombic repulsion [13]. Figure 1B shows the other forces involved in the process of nanofiber formation, such as surface tension, viscoelastic force, air resistance, gravity, and electrostatic force. The electrospinning process starts with the development of the straight jet from the drop forming at the tip of the needle, followed by some instabilities like bending deformation with circular and helical trajectories, ending with the collection of nanofibers on the collector [14].

In addition to electrospinning, there are many other methods of producing nanofibers, including top-down and bottom-up electrospinning, which allow for precise process management and obtaining uniform fibers. Another method is the melt-spinning method, where rapid cooling allows obtaining nanofibers. Another method is coaxial electrospinning, which uses two liquids (e.g., polymer and solution) forced through two separate nozzles, creating fibers with a coaxial structure, as well as others, including force spinning, template synthesis, and drawing [15,16,17]. Overall, although other nanofiber formation methods exist, electrospinning remains the preferred choice for tissue engineering applications due to its versatility, scalability, and ability to produce nanofiber scaffolds with tailored properties to support tissue regeneration.

Electrospun biopolymer scaffolds are extensively investigated in tissue engineering [18,19] to regenerate human systems, such as the nervous [20], cardiovascular, and urinary systems. Artificial polymer bone scaffolds are reported to support bone recovery in the skeletal system [21]. Scientists try to resolve bone tissue engineering (BTE) problems by working with various types of polymers dedicated to BTE [12], mainly using composites of polyesters with HA [22]. Polyesters indicate many various disadvantages. Therefore, PMMA is proposed as a matrix in this paper.

Poly(methyl methacrylate) (PMMA) is a biocompatible polymer, well established in medical applications, with mechanical properties similar to bone [23,24]. PMMA was investigated as a polymer with long-term stability after implantation [25]. In some studies, PMMA was blended with other polymers, such as polypropylene (PP) [26], or with natural rubber to improve compatibility [27]. Pure PMMA has a wide range of biomaterial applications, such as lenses, drug delivery systems, and dental prosthetics [28]. In clinical BTE applications, PMMA is used most often as a component of bone cement [29]. However, in this form, PMMA causes mild damage to bone surrounding tissue [30]. Furthermore, electrospun PMMA scaffolds offer tunable properties such as fiber diameter, porosity, and surface chemistry, which can be tailored to specific BTE requirements. The controlled release of bioactive molecules or growth factors can also be achieved by incorporating them into the electrospun fibers, facilitating tissue regeneration. Additionally, the ease of fabrication and scalability of electrospinning make it a viable option for producing large-scale, customized scaffolds for clinical applications [31]. Therefore, PMMA in the form of electrospun fibers is proposed in this paper for BTE applications.

Natural bone is composed mainly of calcium phosphate (CP, 45–70 wt%), water (10%), and natural collagen [32]. During the bone-forming process, CP mineralization occurs, which leads to the formation of hydroxyapatite (Ca_5_(PO_4_)_3_ OH) [33]. Moreover, minerals such as sodium (Na^+^), hydrogen phosphate (HPO_4_^2−^), carbonate (CO_3_^2−^), and others [34] are utilized in the reaction of bone metabolism. Hydroxyapatite is a bioactive and stable CP mineral at pH, temperature, and composition of intravascular fluid [35]. Thus, hydroxyapatite particles’ (nHA) presence and scaffold composition raise osteoconductivity and bioactivity, and these particles can be used as a filler in bone defects [36].

In this study, composites of PMMA/nHA in the form of nanofibers (low- and high-oriented) were fabricated via the electrospinning process. The aim of the research was to develop material suitable for BTE and optimization of morphology, wettability, chemical structure, biocompatibility, etc. An additional aim was to indicate the influence of fiber alignment on the listed characteristics.

## 2. Materials and Methods

### 2.1. Materials

Poly(methyl methacrylate) (PMMA, Mw = 100,000 g/mol, PMMA 100 K) was purchased from Polysciences, Inc., USA. Hydroxyapatite, a nanopowder with <200 nm particle size, was purchased from Sigma-Aldrich, Germany. N, N-dimethylformamide (DMF) was used as a solvent for dissolving PMMA and was bought from Sigma-Aldrich, Germany. All chemical reagents were analytically pure.

Reagents for in vitro tests, such as amino acids, L-glutamine, fetal bovine serum (FBS), and antibiotics (penicillin/streptomycin), were purchased from Sigma Aldrich (Dorset, UK). Phosphate-buffered saline (PBS), Presto Blue, Trypsin EDTA, Dulbecco’s Modified Eagle Medium, ActinGreen, and NucBlue were purchased from Thermo Fisher Scientific (Waltham, MA, USA). The cell line osteoblasts were purchased from Sigma-Aldrich (Dorset, UK).

### 2.2. Preparation of PMMA/n-HA Solution and Scaffold Fabrication via Electrospinning Technique

Poly(methyl methacrylate) (PMMA) was dissolved in DMF at a concentration of 30 (*w*/*v*%) using a magnetic stirrer set at a rotating speed of 1000 rpm for 8 h. Next, an appropriate amount of hydroxyapatite nanoparticles (nHA) was added to obtain 10 (*w*/*v*%). To avoid nHA aggregation, the solutions were put in an ultrasonic cleaner for 30 min.

Formation of the scaffolds using the electrospinning technique was performed in an electrospinning chamber with controlled parameters (Fluidnatek LE-50, Bioinicia, Valencia, Spain). The scheme of the electrospinning process of nanofibrous scaffolds can be found in our previous work [37]. Several parameters were fixed: process temperature 23 °C, voltage 10 kV, collector rotational speed at 100 and 1000 rpm, solution flow rate 1 mL/h. Electrospinning of the fibers was realized in a horizontal setup position. Next, scaffolds were deposited under a fume hood for 72 h to remove the solvent residue.

Analysis of the SEM images revealed that samples collected at 100 rpm showed slight fiber orientation; however, for simplicity, they were labeled as random. On the other hand, samples collected at 1000 rpm, with high fiber orientation, were labeled as aligned. Samples with the addition of hydroxyapatite nanoparticles were additionally labeled with nHA. When necessary, supplementary information is given in the description of figures or in the text.

### 2.3. Characterization of PMMA/nHA Scaffolds

#### 2.3.1. Morphology Analysis

Morphology analysis was performed using scanning electron microscopy (SEM) imaging (SEM, JSM-6010PLUS/LV InTouchScope™, JEOL, Tokyo, Japan). Before imaging, each nonwoven scaffold was coated with gold. The accelerating voltage was 10 kV. Data analysis was performed using ImageJ 1.52q software version. The fiber diameter distribution was determined using a Gaussian approximation with 100 measurements per sample [38]. Fiber orientation distribution was determined using ImageJ software with the orientation plugin. The Pearson VII function was used to approximate the orientation distribution characterized by FWHM.

The presence of nHA was confirmed using energy-dispersive X-ray spectroscopy (EDS) (JSM-6010PLUS/LV InTouchScope™, JEOL, Tokyo, Japan). Samples were measured using the following parameters: 8 kV, WD = 10, 500 pA, and collecting time 30 min. The EDS results with the distribution of phosphorous (P), oxygen (O), carbon (C), and calcium (Ca) could be observed.

#### 2.3.2. Water Contact Angle Measurements and Surface Tension Energy Determination

The wettability of all nonwoven scaffolds was measured using the water static contact angle method. The investigation was conducted using a Data Physics OCA 15EC contact angle goniometer (Filderstadt, Germany). A distilled water droplet (2 µL volume) was placed on the surface of the scaffolds at room temperature. The water contact angle was calculated after 3 s. Ten measurements were performed for every sample, and the results were averaged.

Surface free energy (SFE) was determined using the Kaelble–Owens–Wendt method, assuming that it is a sum of two main independent components related to dispersion and polar interactions. To evaluate the surface free energy, three liquids were used—diiodomethane with a high dispersive component, polar water, and formamide, as previously described [39]. The values of components $\gamma_{s}^{d}$ and $\gamma_{s}^{p}$ (Equations (1) and (2)) of the tested materials can be calculated from the following:

(1) $${{(\gamma }_{s}^{d})}^{0.5}=\frac{\gamma_{d}\left(cos\theta_{d}+1\right)-\sqrt{\left(\frac{\gamma_{d}^{p}}{\gamma_{w}^{p}}\right)}\gamma_{w}(cos\theta_{w}+1)}{2(\sqrt{\gamma_{d}^{d}}-\sqrt{\gamma_{d}^{p}-(\frac{\gamma_{w}^{p}}{\gamma_{w}^{p}})})}$$

(2) $${{(\gamma }_{s}^{p})}^{0.5}=\frac{\gamma_{w}\left(cos\theta_{w}+1\right)-2\sqrt{\gamma_{s}^{d}\gamma_{w}^{d}}}{2\sqrt{\gamma_{w}^{p}}}$$

where $\gamma_{s}^{d}$ is the dispersion element of the surface free energy of the tested materials, and $\gamma_{s}^{p}$ is the polar element of the tested materials; $\gamma_{d}$— the surface free energy of diiodomethane; $\gamma_{d}^{d}$—the dispersive element of diiodomethane surface energy; $\gamma_{d}^{p}$—the polar element of diiodomethane; $\gamma_{w}$—the surface free energy of water; $\gamma_{w}^{d}$—the dispersive element of water surface free energy; $\gamma_{w}^{p}$—the polar element surface free energy of water; $\theta_{d}$ and $\theta_{w}$—contact angles of diiodomethane and water.

Wu’s method can be used to calculate the surface polarity (*X_p_*) (Equation (3)) [40]:

(3) $$X_{p}=\frac{\gamma_{s}^{p}}{\gamma_{s}}$$

#### 2.3.3. Fourier Transform Infrared Spectroscopy (FTIR)

Molecular structure analysis with the determination of phase content, including the content of the hydroxyapatite addition, was performed using Fourier transform infrared spectroscopy (Bruker Vertex 70, Mannheim, Germany). The data presented are representative of five independent samples and runs. The samples were scanned from 400 to 4000 cm^−1^ with a resolution of 2 cm^−1^ and a total of 32 scans.

#### 2.3.4. Differential Scanning Calorimetry (DSC)

Thermal analysis of PMMA scaffolds, both pure and with nanohydroxyapatite particles, was performed using a differential scanning calorimeter (DSC, Pyris 1, Perkin Elmer, Waltham, MA, USA) equipped with Intracooler 2P under nitrogen atmosphere. Oriented and random fibrous samples (9–11 mg) were loaded into standard aluminum pans. Samples were analyzed in the heating–cooling–heating mode at a rate of 10 K·min^−1^ in the temperature range from 0 °C to 210 °C. For statistics, at least five samples were tested.

It is quite obvious that only the 1st heating scan reflects the state of the as-spun fibers, and the cooling and subsequent 2nd heating scans reflect the state of the sample after the thermal history has been erased. However, quantitative analysis of the 1st heating scans was found difficult, since, besides the PMMA fibers’ glass transition, they show additional thermal effects related to their thermal history. In order to make the analysis of the 1st heating scans feasible, a procedure using the cooling and 2nd heating scans was employed.

Firstly, the baseline-subtracted heating–cooling–heating scans were corrected for the heat flow asymmetry in the cooling–heating modes using a 5th-order polynomial approximation of the heat flow (in W/g) vs. temperature (in °C) as registered for the samples after the thermal history was erased, i.e., during the cooling and 2nd heating scans. Then, the heat flow was normalized with respect to the heating/cooling rates (in K/min) resulting in the heat flow in heat capacity units, i.e., J/gK vs. T (°C) for the whole heating–cooling–heating cycle. This resulted in the superposition of the heat flow during the 1st heating, cooling, and 2nd heating scans at ranges with no thermal effects. This way, the thermal history effects as seen on the 1st heating scans could be determined.

Secondly, the quantitative analysis of all the scans was performed using the “Two-state, two (time)scale model” (TS2) proposed by Ginzburg [41] for a mathematical description of the equilibrium dynamic behavior of amorphous glassy materials. The TS2 model was used for the description of the glass transition during the 1st heating, cooling, and 2nd heating scans, excluding the thermal history effects, under the assumption of linear temperature dependence of the heat flow below and above the glass transition (Equation (4)):

(4) $$HF\left(T\right)=\left(a_{1}+b_{1}\times T\right)\times p_{1}\left(T\right)+\left(a_{2}+b_{2}\times T\right)\times [1-p_{1}\left(T\right)]$$

where *HF(T)*—heat flow; $a_{1}$, $b_{1}$ and $a_{2}$, $b_{2}—$coefficients of the linear *HF(T)* dependence below and above the glass transition, respectively. The characteristics of the HF change through the glass transition are reflected by the weight contribution of the *p1(T)* factor expressed as follows (Equation (5)):

(5) $$p_{1}\left(T\right)=\frac{exp[\frac{∆S}{R}\left(\frac{T_{g}}{T}-1\right)]}{1+exp[\frac{∆S}{R}\left(\frac{T_{g}}{T}-1\right)]}$$

where $\frac{∆S}{R}$ is transition entropy and *T_g_* is the glass transition temperature.

Coefficients of the linear *HF(T)* dependence below and above the glass transition, $a_{1}$, $b_{1}$ and $a_{2}$, $b_{2}$, respectively, as obtained for the cooling and 2nd heating scans served in the glass transition approximation during the 1st heating scans. The latter are presented in the results section along with the thermal history effect data.

#### 2.3.5. Wide Angle X-ray Scattering (WAXS)

Investigations were performed using a Bruker D8 Discover Diffractometer. Measurements were conducted using Cu Kα radiation of wavelength λ = 1.5406 Å at a voltage of 40 kV and current of 40 mA. Measurements were conducted in reflection mode. Formation of the incident beam was performed using optics consisting of Göbel mirrors, a 1 mm linear slit, and a 2.5° n axial Soller. The WAXS profiles were recorded using a Lynxeye one-dimensional detector preceded by a Ni filter and a 2.5° axial Soller.

#### 2.3.6. In Vitro Study

Biocompatibility. Tests were carried out using the cell line human MG-63 (86051601, Sigma Aldrich). Cells were cultivated in a 25 cm2 flask in a medium consisting of High Glucose Dulbecco’s Modified Eagle’s Medium (DMEM), 10% fetal bovine serum (FBS), 1% antibiotics, and 1% glutamine. Cells were incubated in a 5% CO_2_ environment at 37 ℃. For the detachment of cells from the flask, the cells were washed in PBS. Then, 3 mL of 0.05% trypsin solution was added to the cells, and the flask with cells was placed in the incubator for a few minutes. Following the collection of harvested cells, 10 mL of culture medium was introduced, and centrifugation was performed at room temperature. The pellet was resuspended with a culture medium to obtain the required cell density. Different studies were performed to determine the cellular response to monolithic and Janus nanofibers, including cytotoxicity on extracts and cellular morphology.

Cellular viability. An MG-63 cell suspension was seeded on samples and a tissue culture plates (TCP) with a density 1 × 10^4^ cells/well and put in an incubator. After 3 days and 5 days, the culture medium was removed, and each well was filled with 180 mL of PBS and 20 mL of Presto Blue reagent. After this step, the plate was returned to the incubator for 60 min. This procedure was executed, and 100 mL from each well was transferred into a 96-well plate. The fluorescence read with excitation/emission 530/620 nm filters was measured using 530/620 nm excitation/emission wavelength by Fluorescent Accent FL Thermo Fisher Scientific. The results were compared with the Presto Blue fluorescence of blank samples, which did not show metabolic activity, and the control (tissue culture plate (TCP)), which showed 100% viability.

Fibroblast morphology. Verification of cellular morphology in direct contact was performed using fluorescence microscopy. MG-63 cells were seeded on electrospun membranes, and after 3 and 6 days of cultivation, cells were fixed in 3% formaldehyde for 20 min. Then, fixed cells were kept in 0.01% Triton X 100 for 5 min to permeabilize cell membranes. Finally, cellular nuclei and cytoskeleton were stained for 30 min in a mixed solution of ActinGreen and NucBlue, the molecules of which bind to the cytoskeleton and nucleus DNA, respectively. Images were taken with a Leica AM TIRF MC microscope (Germany).

### 2.4. Statistical Analysis

The data were also used to evaluate their statistical significance. The statistical analysis of viability data was conducted for *p* < 0.05 using GraphPad Prism 8.0.1 Software. Two-way ANOVA with Tukey’s multiple comparisons was performed as required. Statistical significance was determined by evaluating the *p*-values. A *p*-value less than 0.05 was regarded as indicating statistical significance. In cases where the *p*-value fell within the range of 0.01 to 0.05, it was denoted as “*”.

## 3. Results and Discussion

### 3.1. Morphology Analysis

**Figure 2:**
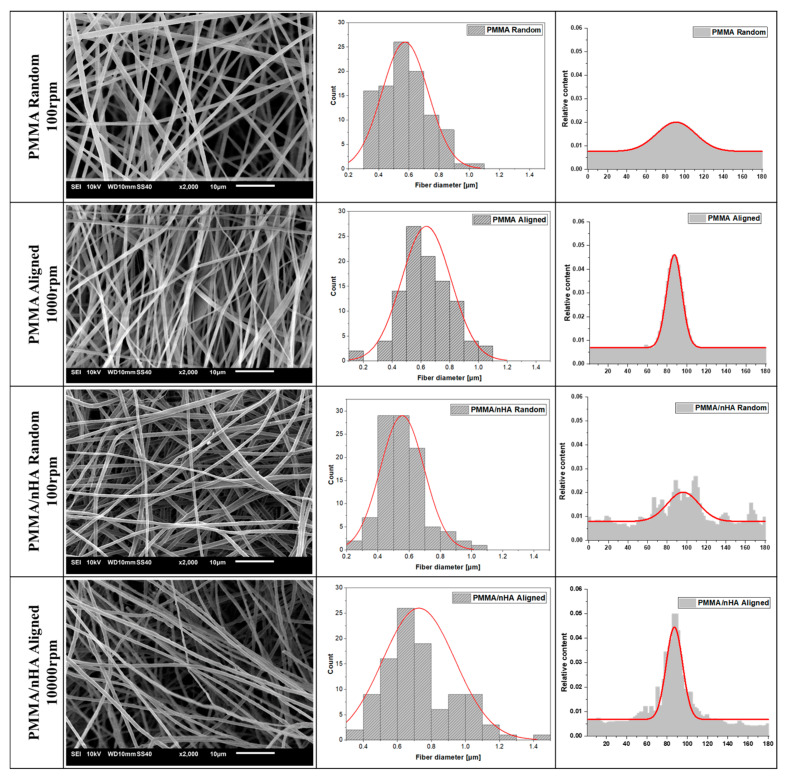
SEM microstructures of pure PMMA and doped with nHA with fiber diameter and orientation distributions (with numerical Gaussian approximation).

SEM analysis indicates the formation of no-bead fiber morphology irrespective of the applied parameters (Figure 2). The thickness of the fibers is higher for the random orientation of fibers: 726.72 ± 21 nm for pure PMMA R and 635.45 ± 17.18 nm in PMMA/nHA R. The higher rotational speed of the collector (1000 rpm) resulted in a considerable decrease in fiber diameter in aligned fiber samples (PMMA A 595.05 ± 18.51 nm and PMMA/nHA A 556.07 ± 14.07 nm).

From the results obtained, it may be inferred that the addition of nHA slightly reduces the average fiber diameter. This is in contrast to the literature data showing no effect of the addition of hydroxyapatite on the fiber diameter, e.g., [42]. It is evident from SEM microstructures that the specimens doped with hydroxyapatite nanoparticles showed uniform nHA distribution inside the fibers with no agglomerations.

From Figure 2, the appearance of fiber orientation at a higher rotational speed of the collector can be seen. The full width at half maximum (FWHM) of the fiber orientation distribution changes from 81.30 for 100 rpm to 27.13 for 1000 rpm for both pure and doped PMMA.

**Figure 3:**
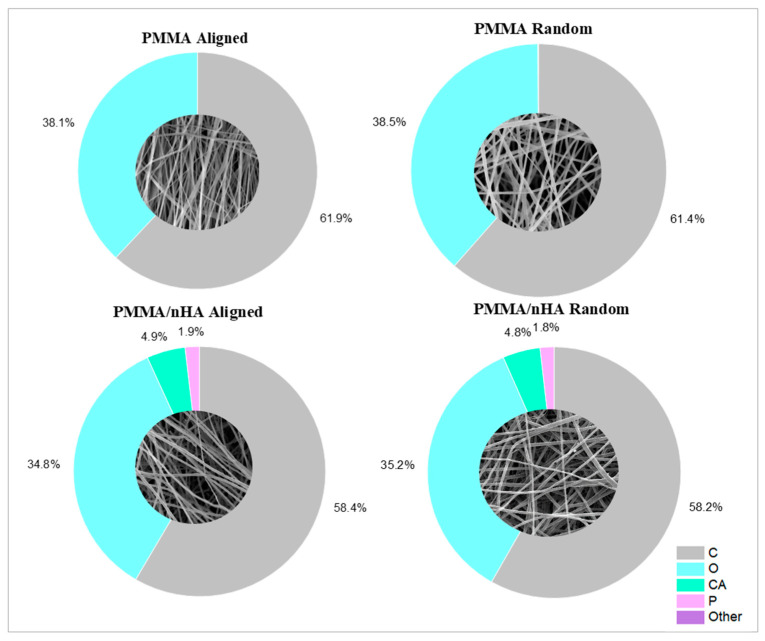
Scaffolds’ surface elemental composition.

The presence of hydroxyapatite nanoparticles was confirmed by energy-dispersive X-ray spectroscopy (EDS) (Figure 3). For doped samples, the presence of the main chemical elements of HA nanoparticles, such as Ca and P, along with the main elements of PMMA—carbon and oxygen—were registered. Measurements using the EsB detector showed material contrasts where differences in grayscale in the sample structure allowed the identification of various material components. Additionally, bright spots on the images in Figure 3 indicate hydroxyapatite nanoparticles, as Ca and P elements are heavier as compared to C and O in the PMMA fibers (Table 1).

### 3.2. Contact Angle Measurements for Surface Free Energy Determination

**Figure 4:**
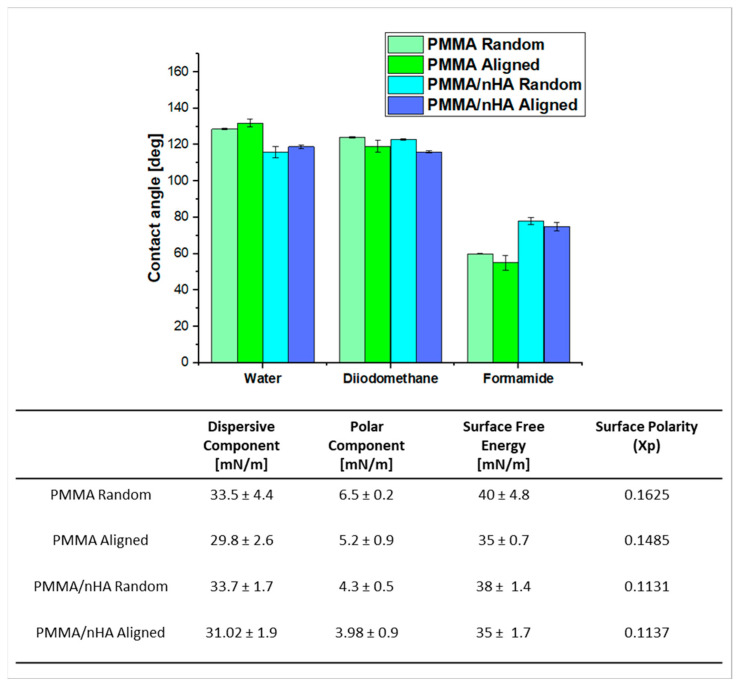
Contact angle measurements, SFE, and their components for all electrospun scaffolds (*p* < 0.05).

Contact angle measurements were performed using water, diiodomethane, and formamide (Figure 4). For water, the contact angle was higher for pure PMMA, both for lower and higher orientations (130° ± 0.3°). Doping with nHA reduced the water wettability of the scaffolds to 125° ± 0.7°. In the case of diiodomethane, the contact angle was comparable to the water contact angle. However, in the case of formamide, the angle for pure PMMA nonwovens was lower, approximately 60°. The addition of nHA increased the contact angle to 80°.

It can be observed that surface free energy (SFE) is generally higher in random samples, being 40 and 38 mN/m for random PMMA and PMMA/nHA, respectively, and 35 mN/m for aligned PMMA, pure and doped with nHA. The SFE decreasing order is as follows: PMMA random > PMMA/nHA random > PMMA aligned > PMMA/nHA aligned (Figure 4). The Kaelble–Owens–Wendt method was used for the calculation of the total SFE as a sum of the polar component due to hydrogen bonds and dipole–dipole interactions and a dispersive component due to London and van der Waals forces [39]. It was observed that the dominant factor contributing to the SFE of the PMMA substrates was the dispersive component. Furthermore, the dispersive component exhibited a decreasing trend as follows: PMMA/nHA random > PMMA random > PMMA/nHA aligned > PMMA aligned.

Additionally, the polar component of SFE for random fibers was slightly higher than for aligned fibers, which suggests polar contributions to surface interaction The presence of HA weakens the polar interactions of PMMA [43].

### 3.3. Fourier Transform Infrared Spectroscopy (FTIR)

**Figure 5:**
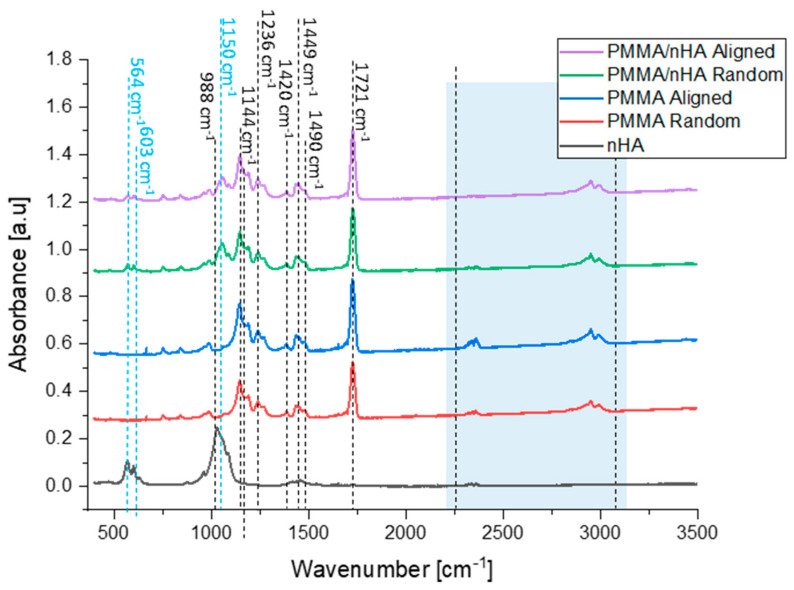
FTIR spectra of hydroxyapatite particles, pure PMMA, and PMMA/nHA scaffolds with random and aligned fiber orientation.

In order to investigate the structural effects of nHA addition on PMMA fibers, FTIR measurements were performed. The spectra are presented in Figure 5.

PMMA bands of the ester carbonyl group C=O at 1721 cm^−1^ and C-O-C stretching at 1144 cm^−1^ were observed in all nanofibrous samples [44]. The peaks registered at 1420 cm^−1^, 1449 cm^−1^, and 1490 cm^−1^ are bending vibrations of OCH_3_, CH_3_, CH_2_ groups [45]. Further, the peak at 1236 cm^−1^ is a stretching vibration of C-O-C groups, while the peak at 988 cm^−1^ is a C-O-CH_3_ group rocking vibration. Peaks at 564 cm^−1^ and 603 cm^−1^ are identified as a symmetric stretching vibration of P-O of the hydroxyapatite and are observed in samples containing nHa in random and aligned orientations [46]. Additionally, one peak at 1150 cm^−1^ can be assigned to the presence of P-O [47,48]. Therefore, hydroxyapatite nanoparticles were successfully mixed into the PMMA scaffolds, which is in agreement with SEM-EDS results. PMMA chains associated with nHA are responsible for additional vibrations in particular wavelengths, namely 2250–2300 cm^–1^ (C=O) and 2700–3000 cm^–1^ (O–CH_3_) [35,49,50] (Table 2).

### 3.4. Differential Scanning Calorimetry (DSC)

**Figure 6:**
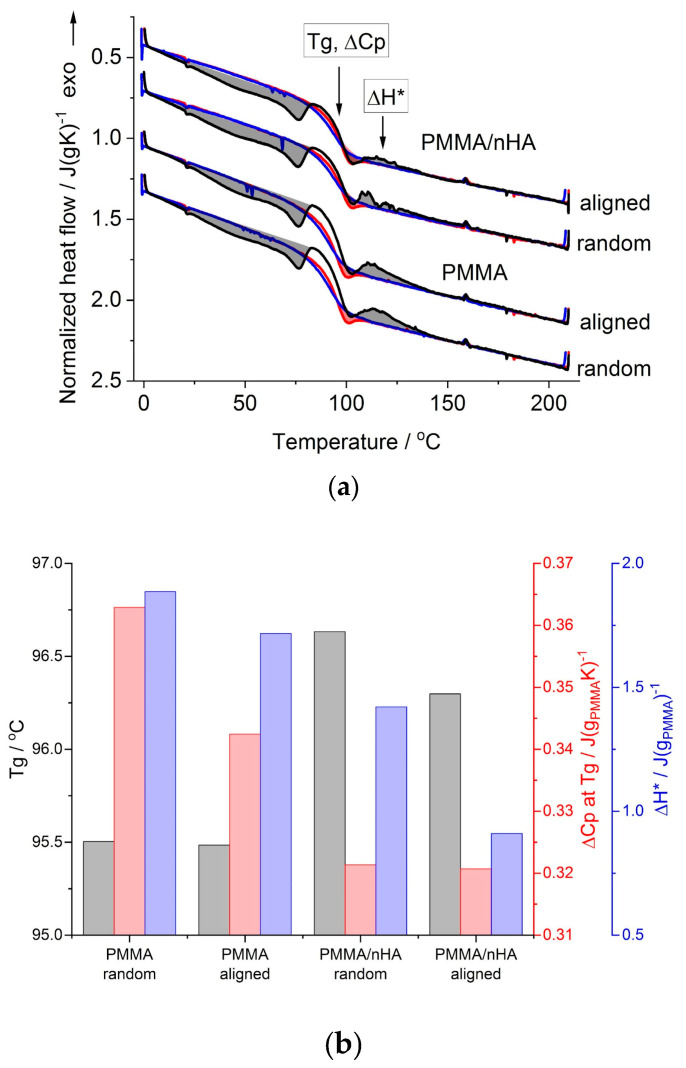
DSC results for random and aligned PMMA fibers, pure and with nHA: (**a**) 1st heating (black lines), cooling (blue lines) and 2nd heating (red lines) scans; shaded areas are the thermal effects observed during 1st (grey-colored) and 2nd (red-colored) heating scans (see the text); (**b**) glass transition temperature, *Tg*; heat capacity change at *Tg*, *ΔCp* at *Tg*; heat of the exothermic effect, ΔH*, as determined from the 1st heating scans.

DSC results are presented in Figure 6, showing the first heating, cooling, and second heating scans (Figure 6a) registered for random and aligned fibers spun from pure PMMA and PMMA with nanohydroxyapatite particles (PMMA/nHA). The first heating scans (black curves in Figure 6a) start with a broad endothermic effect with a local maximum at ca. 75 °C. This effect is followed by a step increase in the heat flow in the temperature range of 90–100 °C, ending with a small endothermic overshoot, followed by an exothermic effect at ca. 110 °C. These endo- and exothermic effects on the first heating scans were grey-shaded. Above the exothermic effect, there is a lack of any endothermic effects in the upper temperature range up to 210 °C, which indicates a lack of crystallinity, or in other words, the amorphous state of PMMA in all of the samples.

On the cooling scans (blue curves in Figure 6a), there appears only a step change in the heat flow, seen in the temperature range of 80–100 °C. A similar step change in the heat flow is present on the second heating scans (red curves in Figure 6a), but at the end of this step, ca. at 100 °C, there is an overshoot in the heat flow, marked by a red-colored shaded area.

The step change in the heat flow, seen on all the heating and cooling scans (Figure 6a), is related to the change in PMMA heat capacity during the glass transition. This range is annotated in Figure 6a as the glass transition temperature, *Tg*, with a change in the heat capacity, *ΔCp*. The cooling and second heating scans, which reflect the state of the samples after the thermal history is erased, serve as the reference, which enables the differences in the glass transition range and the thermal history effects appearing during the first heating scans, which reflect the state of the as-spun fibers, to be revealed. Thus, the cooling and second heating scans were used as a reference in the quantitative analysis of the first heating scans with the TS2 [36] model, provided earlier in the experimental section.

The values of the *Tg* and *ΔCp* determined from the first heating scans are shown in Figure 6b. It is seen that for PMMA fibers doped with nHA, the *Tg* is higher by more than 1 deg and the *ΔCp* is lower than that for pure PMMA fibers. The effect of fibers’ alignment is seen as lower *Tg*, especially in PMMA/nHA fibers, and as lower *ΔCp*, especially in pure PMMA fibers, in reference to random fibers. Higher *Tg* and lower *ΔCp* in the case of PMMA/nHA fibers may be described as the stiffening effect of nHA particles. In the case of aligned fibers, lower *Tg* may be explained as being due to higher stress accumulated during the drawing of the fibers collected at 1000 rpm resulting in much easier stress relaxation in more strained polymer chains. The exothermic effect above the glass transition (grey-shaded and marked with ΔH* in Figure 6a) is most probably also a manifestation of the stress relaxation accumulated in fibers during electrospinning. The heat of this effect was found to be lower in PMMA/nHA vs. PMMA fibers and in aligned vs. random fibers (Figure 6b). The former effect may be explained by increased stiffness in PMMA/nHA fibers, where nHA particles may act as additional entanglement sites, which reduce macromolecules’ stress relaxation ability. The latter effect may be explained as due to a higher ratio of strained vs. mobile chain segments between entanglement sites, resulting in lower stress relaxation ability of strained chains in aligned fibers.

Additionally, the low-temperature endothermic effect observed with a local maximum at ca. 75 °C during the first heating scan is most probably related to the evaporation of the solvent residue. It is indicated by a similar value of the heat of this effect for pure and doped with nHA samples, being ca. 5 J/g.

### 3.5. Wide Angle X-ray Scattering (WAXS)

**Figure 7:**
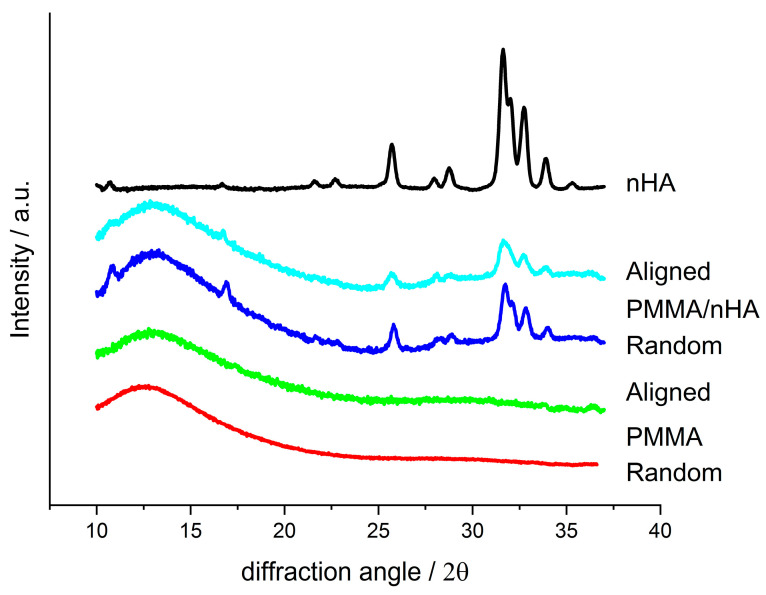
WAXS profiles registered for random and aligned fibers made of pure PMMA and PMMA with addition of nHA and for pure nHA.

Figure 7 presents WAXS profiles registered for random and aligned PMMA fibers, pure and doped with nHA; a profile for pure nHA is also presented for comparison. On the profiles for doped fibers, the presence of nHA diffraction peaks is seen as similar to the nHA reference profile. For all the fibrous samples, the profiles show an amorphous halo located at 2θ ca. 12.8° for pure PMMA and at 13° for PMMA/nHA fibers. There are no additional diffraction peaks, which indicates that PMMA is in the amorphous state. Moreover, there is no particular difference between the profiles for the random and aligned fibers.

### 3.6. In Vitro Results

Osteoblasts are fundamental for continuous bone remodeling and play an important role in the evaluation of materials for BTE [52]. The MG63 cell line is a human osteoblastic line recommended by the International Organization for Standardization (ISO) for assessment of biocompatibility in 10993-5 Biological evaluation of medical devices, Part 5: Tests for in vitro cytotoxicity. Hence, viability was determined on sample extracts, as in a previous study [53]. Additionally, MG-63 cells are used in osteogenesis studies because they remain stable in their phenotype over several passages in cell culture. Therefore, parallel to cellular viability, we determined morphology and cell–material interaction.

**Figure 8:**
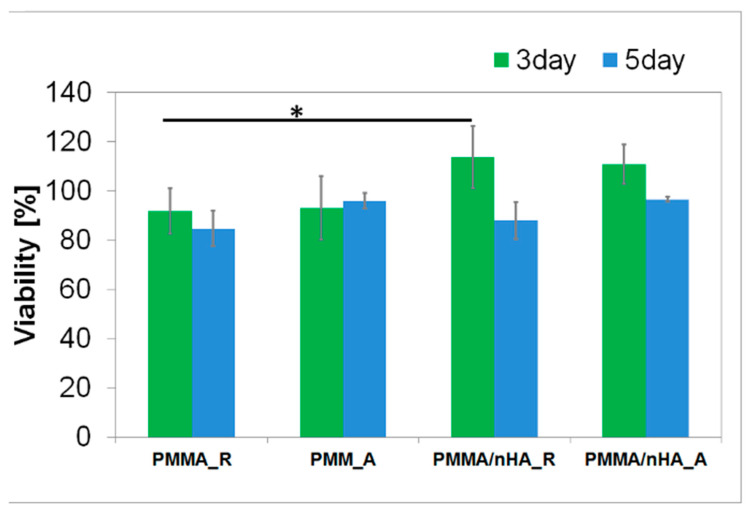

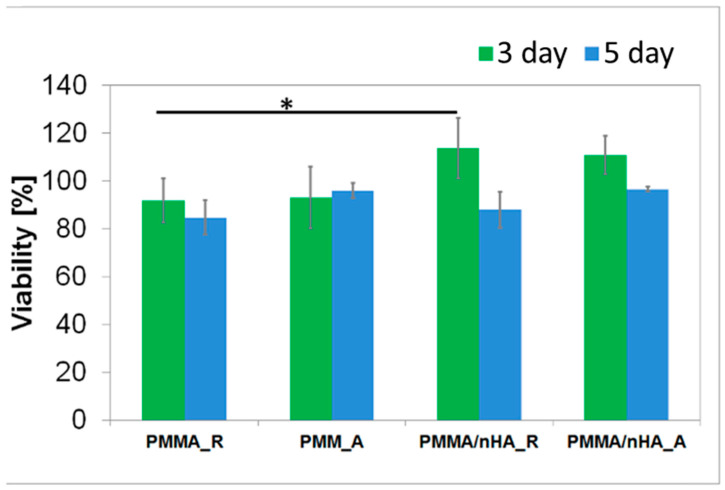
Cellular viability in contact with PMMA random (PMMA_R), PMMA aligned (PMMA_A), PMMA/nHA random (PMMA/nHA_R), and PMMA/nHA aligned (PMMA/nHA_R) composites as the rate of TCP (Tissue Culture Plastic, 100%). The data are presented as mean with standard deviation and were found to be statistically insignificant. Statistical significance: * *p* < 0.05.

In vitro, direct contact of MG63 cells with extracts of pristine PMMA scaffolds and PMMA/nHA scaffolds was evaluated (Figure 8). All samples reached values ≥ 70% of the TCP control after 3 days and 5 days of contact with the sample, which is in agreement with the standard for living cells from ISO 10993-5. All samples meet the requirements of non-toxicity.

**Figure 9:**
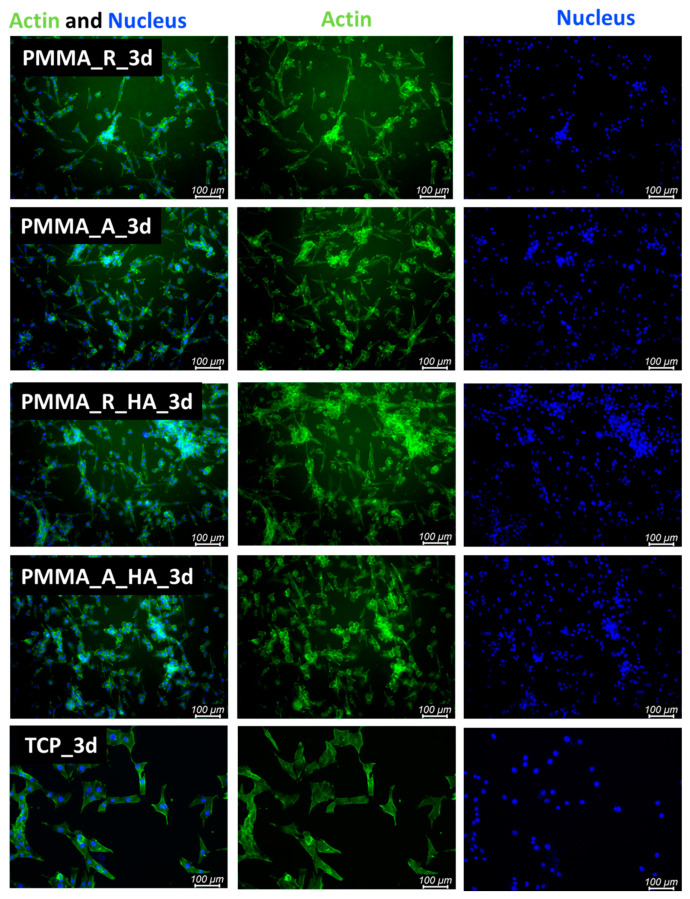
FM images of stained MG-63 directly cultured on the specimen substrate for 3 days. PMMA random (PMMA_R), PMMA aligned (PMMA_A), PMMA/nHA random (PMMA/nHA_R), and PMMA/nHA aligned (PMMA/nHA_R) in comparison to TCP.

**Figure 10:**
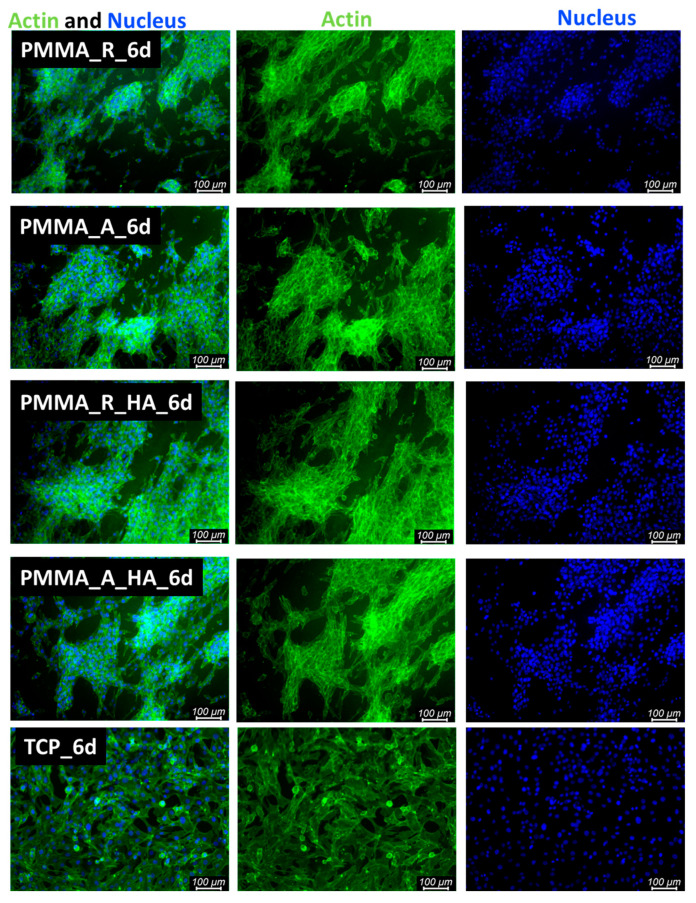
FM images of stained MG-63 directly cultured on the specimen substrate for 6 days. PMMA random (PMMA_R), PMMA aligned (PMMA_A), PMMA/nHA random (PMMA/nHA_R), and PMMA/nHA aligned (PMMA/nHA_R) in comparison to TCP.

Cell proliferation and attachment are the first steps of the tissue regeneration process and a prerequisite for the growth of the cells [54]. Therefore, cellular morphology was evaluated. After 3 days and 6 days of cultivation of cells seeded in direct contact on pristine PMMA scaffolds and PMMA/nH, cellular morphology was illustrated with fluorescence microscopy (Figure 9 and Figure 10). The osteoblasts’ amount and shape in contact with the sample’s surface were similar to the TCP, which corresponds with cellular viability. The cells were well spread and exhibited good intercellular interactions. Suitable cell–material interaction was confirmed by cell filopodia and lamellipodia elongation and their attachment to the membrane surface. The cells exhibited good spreading on PMMA/nHA composite nanofibrous. Obviously, previous research indicated that it is very important to incorporate nanosized components like nHA in engineered scaffolds in order to stimulate cell proliferation [55]. Consequently, the better adhesion and proliferation of PMMA/nHA fibers might be attributed to the addition of nHA nanoparticles. This in vitro test demonstrated that the PMMA/nHA composite scaffold was more bioactive and had the potential to activate bone formation [56].

## 4. Conclusions

Firstly, we have demonstrated the high spinnability of PMMA/nHA fibers, confirming the thorough incorporation of nHA particles within nanofibers by SEM-EDS and FTIR-ATR methods.

Secondly, the DSC analysis shows that the addition of nHA (1) increases the PMMA glass transition temperature, Tg; (2) reduces the PMMA heat capacity change at Tg, ΔCp; and (3) reduces the relaxation effect of the stress accumulated during electrospinning (seen as the exothermic effect above the glass transition). These effects indicate increased stiffness in PMMA/nHA fibers, where nHA particles may act as additional entanglement sites. This is most probably due to increased interfacial interactions between nHA and PMMA. A similar effect was observed by Ken-Hsuan Liao et al. [14] for composites of PMMA with graphene. Thus, it can be explained by higher polymer matrix immobilization (increased Tg caused by PMMA interface conformation, changes in interaction density between PMMA and nHA) at the hydroxyapatite interface and covalent bonds between PMMA and nHA.

Moreover, for aligned fibers, both pure PMMA and PMMA doped with nHA, the DSC results show lower Tg and lower ΔCp. This may be explained as due to a lower energetical barrier for stress relaxation in more strained polymer chains. On the other hand, the reduced relaxation effect of the stress accumulated by the chain segments during fiber electrospinning/drawing in aligned fibers is explained as due to a higher ratio of strained vs. mobile chain segments between entanglement sites, resulting in a lower stress relaxation ability of strained chains in aligned fibers.

Hydroxyapatite is highly hydrophilic [57], but the water contact angle of PMMA/nHA did not decrease significantly in comparison to pure PMMA. Apart from the possible instrumental effects, one of the possible non-instrumental reasons is related to the low content of nHA particles on the fiber surface [58]. The SFE of the aligned fibers, for both pure PMMA and PMMA/nHA composites, is slightly lower than the SFE of the random fibers. Moreover, nHA nanoparticles promoted the bioactivity of the fibrous scaffold. Thus, PMMA/nHA was equipped with better bioactivity. Future research is needed to validate bone defect treatment in vivo.

## Figures and Tables

**Table 1:** Scaffold elemental composition in wt% as measured by SEM-EDS.

Element	PMMA Aligned	PMMA Random	PMMA/nHA Aligned	PMMA/nHA Random
C	61.91 ± 0.6	61.43 ± 0.4	58.42 ± 0.6	58.20 ± 0.7
O	38.08 ± 0.7	38.45 ± 0.2	34.80 ± 0.3	35.22 ± 0.5
Ca	-	-	4.93 ± 0.5	4.77 ± 0.7
P	-	-	1.85 ± 0.2	1.80 ± 0.2
Other	0.01 ± 0.001	0.11 ± 0.05	-	-

**Table 2:** Wavenumbers and vibrational modes exhibited by samples.

Type of Vibration	Wavenumber [cm^−1^]	References
--OCH_3_ stretching	1195	[45,47]
C=O stretching	1144, 1700–1744	[51]
CH_3_ stretching	1420–1490	[52]
C-H stretching	2930–2986	[52]

## Data Availability

The raw data supporting the conclusions of this article will be made available by the authors on request.
